# Efficacy and prognostic analysis of chemo-immunotherapy after TKI resistance in EGFR-mutant non-small cell lung cancer with TP53 or KRAS co-mutations

**DOI:** 10.3389/fimmu.2025.1684089

**Published:** 2025-11-11

**Authors:** Yuhui Nie, Chen Song, Kun Wu, Mingxin Yu, Jia Hu, Shuzhen Liu, Fu Hui

**Affiliations:** 1 Weifang People’s Hospital, Shandong Second Medical University, Weifang, Shandong, China; 2 School of Clinical Medicine, Shandong Second Medical University, Weifang, Shandong, China; 3 Department of Oncology, Shandong Provincial Key Medical and Health Discipline, Qingdao Central Hospital, University of Health and Rehabilitation Sciences, Qingdao, China

**Keywords:** non-small cell lung cancer, EGFR-TKI, co-mutation, immunotherapy, progression-free survival

## Abstract

**Objective:**

To investigate the impact of co-mutations of EGFR with TP53 or KRAS on the prognosis of non-small cell lung cancer (NSCLC) patients, and the efficacy of platinum-based doublet chemotherapy plus immunotherapy after EGFR-TKI resistance.

**Methods:**

This was a retrospective study that included 168 patients with locally advanced or advanced NSCLC who had next-generation sequencing (NGS) performed at our institution between January 1, 2021, and October 31, 2023. Based on their genomic profiles, patients were categorized into three groups: EGFR single mutation, EGFR/TP53 co-mutation, and EGFR/KRAS co-mutation. Baseline clinical data were collected, including gender, age, smoking history, histological subtype, clinical stage, ECOG performance status, gene testing results, and treatment regimens. All patients were treated with EGFR tyrosine kinase inhibitors (TKIs) as first-line therapy, including first-, second-, or third-generation agents. Upon disease progression, patients received platinum-based doublet chemotherapy plus immunotherapy as second-line treatment. The primary endpoint was progression-free survival (PFS). Survival curves were generated using the Kaplan-Meier method and compared by log-rank test. Baseline characteristics among the three groups were compared using the chi-square test. Multivariate Cox regression analysis was performed to evaluate independent prognostic factors for PFS by incorporating all baseline clinical variables and gene mutation status into the model.

**Results:**

A total of 168 patients were included in the analysis: 36 with EGFR single mutation, 80 with EGFR/TP53 co-mutation, and 52 with EGFR/KRAS co-mutation. There were no statistically significant differences among the three groups with respect to baseline characteristics, including gender, age, smoking history, histological type, clinical stage, and ECOG performance status (*P* > 0.05). Immune-related marker expression was significantly different between the EGFR single mutation group and the two co-mutation groups (*P* < 0.05), while no significant difference was observed between the co-mutation groups (*P* = 0.945). Following first-line EGFR-TKI therapy, the EGFR single mutation group showed a significantly longer median PFS compared with the EGFR/TP53 and EGFR/K-RAS co-mutation groups (*P* < 0.0001). No significant difference in PFS was observed between the two co-mutation groups (*P* = 0.174). Following progression on EGFR-TKIs, all patients received platinum-based doublet chemotherapy plus immunotherapy. In second-line treatment, the median PFS in the EGFR single-mutation group, which was shorter than in the EGFR/TP53 and EGFR/KRAS co-mutation groups (overall log-rank *P* < 0.0001), with no significant difference between the two co-mutation cohorts (*P* = 0.174). However, in multivariable Cox models adjusting for age, sex, smoking history, clinical stage, histology, and ECOG performance status, both EGFR/TP53 and EGFR/KRAS co-mutations were independently associated with a higher hazard of progression. ECOG PS ≥2 was associated with a numerically higher hazard that did not reach statistical significance. No significant associations were observed for other covariates (age, sex, smoking history, clinical stage, histology; all *P*>0.05).

**Conclusion:**

In the first-line setting, patients with an EGFR single mutation treated with EGFR-TKIs had a longer median PFS than those with EGFR/TP53 and EGFR/KRAS co-mutations (14.1 vs 10.4 and 10.9 months, respectively; both *P* < 0.0001), whereas no statistically significant difference was observed between the two co-mutation subgroups (*P* = 0.174). Following the development of resistance, all patients received platinum-based doublet chemotherapy plus immunotherapy; in the second-line setting, median PFS was modestly longer in the co-mutation groups compared with the single-mutation group (EGFR/TP53: 5.2 months; EGFR/KRAS: 5.0 months; EGFR single mutation: 3.9 months; overall log-rank *P* < 0.0001), with no significant difference between the TP53 and KRAS subgroups (*P* = 0.174). These associations were evident on Kaplan–Meier curves (with numbers at risk) and log-rank testing, and were supported by multivariable Cox models adjusted for age, sex, smoking history, clinical stage, histology, and ECOG performance status.

## 1 Introduction

Lung cancer is one of the most prevalent and lethal malignancies worldwide, and it remains the leading cause of cancer-related death (1). The pathological types of lung cancer are mainly divided into small cell lung cancer (SCLC) and non-small cell lung cancer (NSCLC), with NSCLC accounting for approximately 80%, including adenocarcinoma and squamous cell carcinoma (2, 3). Although low-dose spiral computed tomography (CT) can detect lung cancer at an early stage, more than 40% of patients are diagnosed at locally advanced or advanced stages, losing the opportunity for a cure (4). Treatment options for NSCLC are diverse, including surgery, chemotherapy, radiotherapy, targeted therapy, and immunotherapy; however, even in cases of early detection, the majority of patients progress to advanced stages within five years. The 5-year survival rates for locally advanced and advanced NSCLC are less than 15% and 5%, respectively (5).

With advances in genomics and high-throughput gene sequencing, tumor treatment has entered an era of precision medicine. The widespread application of EGFR tyrosine kinase inhibitors (TKIs) has significantly improved the prognosis of NSCLC patients and has become the standard treatment for locally advanced and advanced NSCLC. However, there is significant heterogeneity in the efficacy of EGFR-TKI, with progression-free survival (PFS) ranging from several months to years (6). Some patients exhibit primary resistance to EGFR-TKIs, while others initially respond but ultimately experience divergent outcomes. The mechanisms underlying primary resistance to EGFR-TKIs are complex and may be related to factors such as EGFR-insensitive mutations (e.g., exon 20 insertions, T790M mutations), downstream gene mutations (e.g., KRAS, BRAF, PIK3CA), co-occurring mutations (e.g., TP53, MET, ALK rearrangements), changes in microRNA, and BIM gene deletion (7–12). Among these, the impact of co-mutations involving EGFR and other genes on EGFR-TKI therapy has drawn significant attention.

TP53 is an important tumor suppressor gene that inhibits abnormal cell proliferation by regulating the cell cycle and inducing apoptosis (13), and it is referred to as the “guardian gene.” TP53 mutations can lead to cellular malignancy and increased aggressiveness (14). Currently, there are no targeted drugs available for TP53, and studies have shown that EGFR-mutant NSCLC patients with co-occurring TP53 mutations are more prone to resistance against EGFR-TKIs. Therefore, new treatment strategies are required to improve survival benefits after resistance develops. KRAS gene mutations are common in smokers and lead to sustained activation of GTPase, which results in continuous activation of downstream signaling pathways and, ultimately, cellular malignancy (15). KRAS and EGFR mutations occur independently and are associated with primary resistance to EGFR-TKI therapy (16). For these patients, choosing an appropriate treatment strategy post-resistance is crucial.

## 2 Materials and methods

This study retrospectively analyzed 168 patients with stage III or IV non-small cell lung cancer (NSCLC) who were treated at our hospital’s oncology department between January 1, 2021, and October 31, 2023. The cohort included 36 patients with EGFR mutations alone, 80 with EGFR/TP53 co-mutations, and 52 with EGFR/KRAS co-mutations. Inclusion criteria were: age between 18 and 85 years, histologically confirmed locally advanced or advanced NSCLC, initial treatment with EGFR-TKI, and subsequent chemotherapy plus immunotherapy after EGFR-TKI resistance. Exclusion criteria included incomplete medical history, presence of other malignancies, or inability to undergo further treatment. Data collected included demographic characteristics, tumor staging, and treatment modalities. Efficacy was evaluated based on RECIST 1.1 criteria, with CT scans performed every 3 months to calculate progression-free survival (PFS). Follow-up was conducted until December 31, 2023. Statistical analyses were performed using GraphPad Prism version 9.0. Categorical variables were compared using the chi-square test. Survival outcomes were analyzed using the Kaplan–Meier method, and differences between groups were assessed with the log-rank test. To identify independent prognostic factors for progression-free survival (PFS), all baseline variables were included in a multivariate Cox proportional hazards regression model. A two-sided *P* value < 0.05 was considered statistically significant.

PD-L1 immunohistochemistry was performed on neutral-buffered formalin-fixed, paraffin-embedded (FFPE) tumor specimens using the Dako 22C3 pharmDx assay (clone 22C3). Tumor proportion score (TPS)—the percentage of viable tumor cells with partial or complete membranous staining—was recorded, and PD-L1 expression was categorized *a priori* as negative (TPS <1%), low (1–49%), or high (≥50%). Slides were independently evaluated under blinded conditions (masked to clinical data and outcomes) by two board-certified pathologists; discrepancies—defined as a discordant TPS category or a ≥10-percentage-point difference—were resolved by joint consensus review. Positive and negative control slides accompanied each staining batch, and interpretation proceeded only after quality-control criteria were met.

This study’s retrospective, single-center design may introduce selection bias and limits generalizability; prospective multi-center studies are warranted.

## 3 Results

### 3.1 General clinical data

Baseline clinical characteristics of the three groups are summarized in **Table 1**. Distributions of key variables (age, sex, ECOG performance status, stage, histology, smoking status) were comparable across groups; no between-group differences reached statistical significance (all *P*>0.05).

**Table 1:** Analysis of baseline characteristics among patients with different gene.

Characteristic	Total (n = 168)	EGFR Single Mutation	EGFR with TP53 Co-mutation	EGFR with KRAS Co-mutation	*p*
Gender					0.17
Male	89 (52.98%)	20 (55.56%)	47 (58.75%)	22 (42.31%)	
Female	79 (47.02%)	16 (44.44%)	33 (41.25%)	30 (57.69%)	
Age (years)					0.36
≥60	103 (61.31%)	22 (61.11%)	53 (66.25%)	28 (53.85%)	
<60	65 (38.69%)	14 (38.89%)	27 (33.75%)	24 (46.15%)	
Smoking History					0.307
Yes	111 (66.07%)	25 (69.44%)	56 (70%)	30 (57.69%)	
No	57 (33.93%)	11 (30.56%)	24 (30%)	22 (42.31%)	
Pathological Type					0.076
Adenocarcinoma	155 (92.23%)	30 (83.33%)	76 (95%)	49 (94.23%)	
Non-adenocarcinoma	13 (0.73%)	6 (16.67%)	4 (5%)	3 (5.77%)	
Stage					0.5
Stage III	19 (11.31%)	4 (11.11%)	7 (8.75%)	8 (15.38%)	
Stage IV	149 (88.69%)	32 (88.89%)	73 (91.25%)	44 (84.62%)	
ECOG Score					0.916
0-1	156 (92.86%)	34 (94.44%)	74 (92.5%)	48 (92.31%)	
≥2	12 (7.14%)	2 (5.56%)	6 (7.5%)	4 (7.69%)	

### 3.2 Efficacy of first-line TKI in EGFR single vs. co-mutations

**Figure 1:**
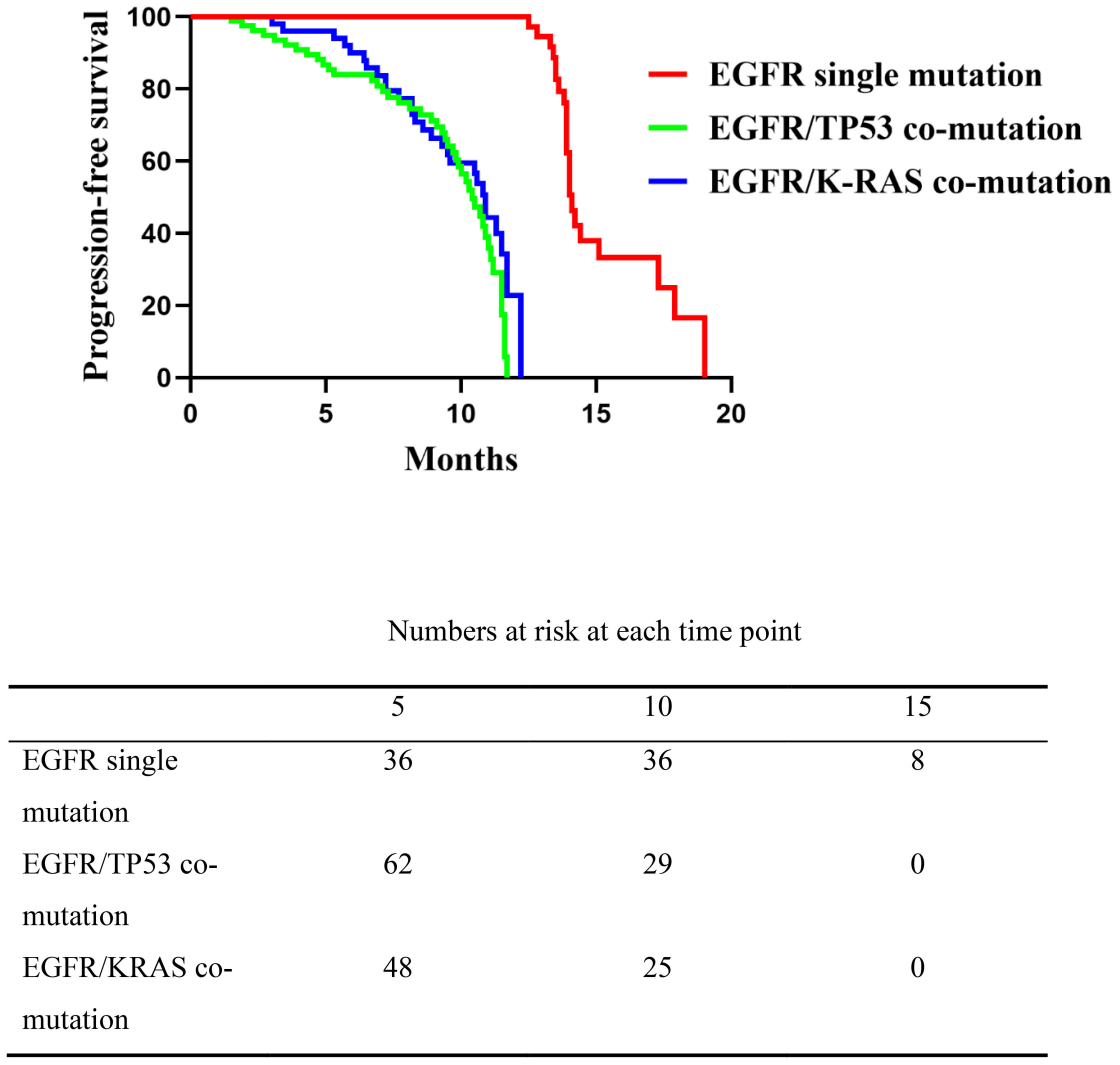
Comparison of progression-free survival (PFS) among the three patient groups receiving first-line EGFR-TKI therapy. The EGFR single mutation group showed significantly longer PFS than the co-mutation groups (*P* < 0.0001), while no significant difference was observed between the EGFR/TP53 and EGFR/K-RAS co-mutation groups (*P* = 0.174).

Patients with an EGFR single mutation had a median PFS of 14.1 months (range, 12.5–19.0), which was longer than in the EGFR/TP53 (median 10.4 months; range, 1.5–11.7) and EGFR/KRAS (median 10.9 months; range, 2.6–12.2) co-mutation groups. The overall log-rank test indicated a significant difference between the single-mutation group and either co-mutation group (*P* < 0.0001), whereas no significant difference was observed between the EGFR/TP53 and EGFR/KRAS cohorts (*P* = 0.174, 95%CI:0.4609-1.162) (**Figure 1**). These results indicate that, compared with the EGFR single-mutation group, the presence of TP53 or KRAS co-mutations was associated with shorter PFS on first-line EGFR-TKI therapy, with no clear difference between the two co-mutation subgroups.

### 3.3 PD-L1 expression in EGFR single vs. co-mutations

PD-L1 expression was categorized by tumor proportion score (TPS) as negative (<1%), low (1–49%), or high (≥50%). Among 168 patients, the overall distribution of PD-L1 categories differed across mutation groups (EGFR single-mutation, EGFR/TP53, EGFR/KRAS) on χ² testing (*P* = 0.011; **Table 2**). In pairwise comparisons, the distributions differed between the EGFR single-mutation group and the EGFR/TP53 group (*P* = 0.004), and between the EGFR single-mutation group and the EGFR/KRAS group (*P* = 0.015), whereas no difference was observed between the EGFR/TP53 and EGFR/KRAS groups (*P* = 0.945). These findings indicate that PD-L1 expression patterns were associated with mutation status; however, no clear difference was seen between the two co-mutation cohorts.

**Table 2:** Analysis of differences in immune expression status among patients with different gene mutation types.

Characteristic	Total (n = 168)	EGFR Single Mutation	EGFR with TP53 Co-mutation	EGFR with K-RAS Co-mutation	*P*
PD-L1					0.011
Negative	16 (9.52%)	8 (22.22%)	5 (6.25%)	3 (5.77%)	
Low Expression	69 (41.07%)	18 (50%)	30 (37.5%)	21 (40.38%)	
High Expression	83 (49.40%)	10 (27.78%)	45 (56.25%)	28 (53.85)	

### 3.4 Efficacy of chemotherapy plus immunotherapy in TKI-resistant EGFR single vs. co-mutations

**Figure 2:**
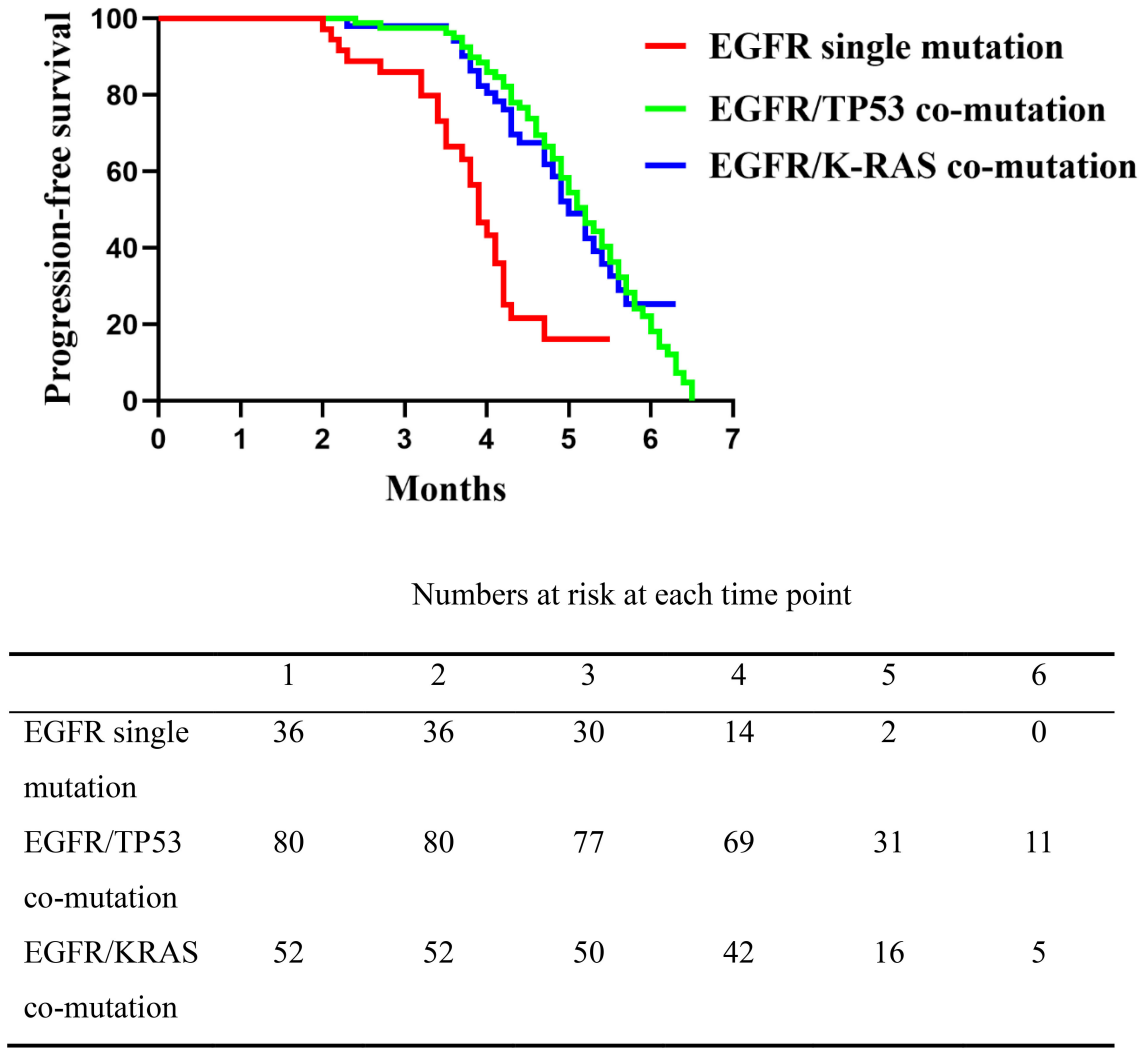
Comparison of progression-free survival (PFS) among the three patient groups following second-line platinum-based doublet chemotherapy combined with immunotherapy. The EGFR single mutation group had significantly shorter PFS compared to the EGFR/TP53 and EGFR/K-RAS co-mutation groups (*P* < 0.0001), while no significant difference was observed between the two co-mutation groups (*P* = 0.1744).

Following progression on EGFR-TKIs, all patients received platinum-based doublet chemotherapy plus immunotherapy. On unadjusted Kaplan–Meier analysis of second-line therapy, median PFS was 3.9 months in the EGFR single-mutation group (range, 2.5–5.5), compared with 5.2 months in the EGFR/TP53 group (range, 2.4–6.5) and 5.0 months in the EGFR/KRAS group (range, 2.3–6.3). The single-mutation group differed significantly from each co-mutation group (overall log-rank *P* < 0.0001) (EGFR single-mutation vs EGFR/TP53 95CI%: 0.1618-0.61), (single-mutation vs KRAS 95%CI: 0.1909-0.6708), whereas no difference was observed between the EGFR/TP53 and EGFR/KRAS cohorts (*P* = 0.943,95%CI: 0.6504-1.587) (**Figure 2**). These results indicate that, relative to the EGFR single-mutation group, the presence of TP53 or KRAS co-mutations was associated with longer PFS on second-line chemo-immunotherapy, with no clear difference between the two co-mutation subgroups.

**Figure 3:**
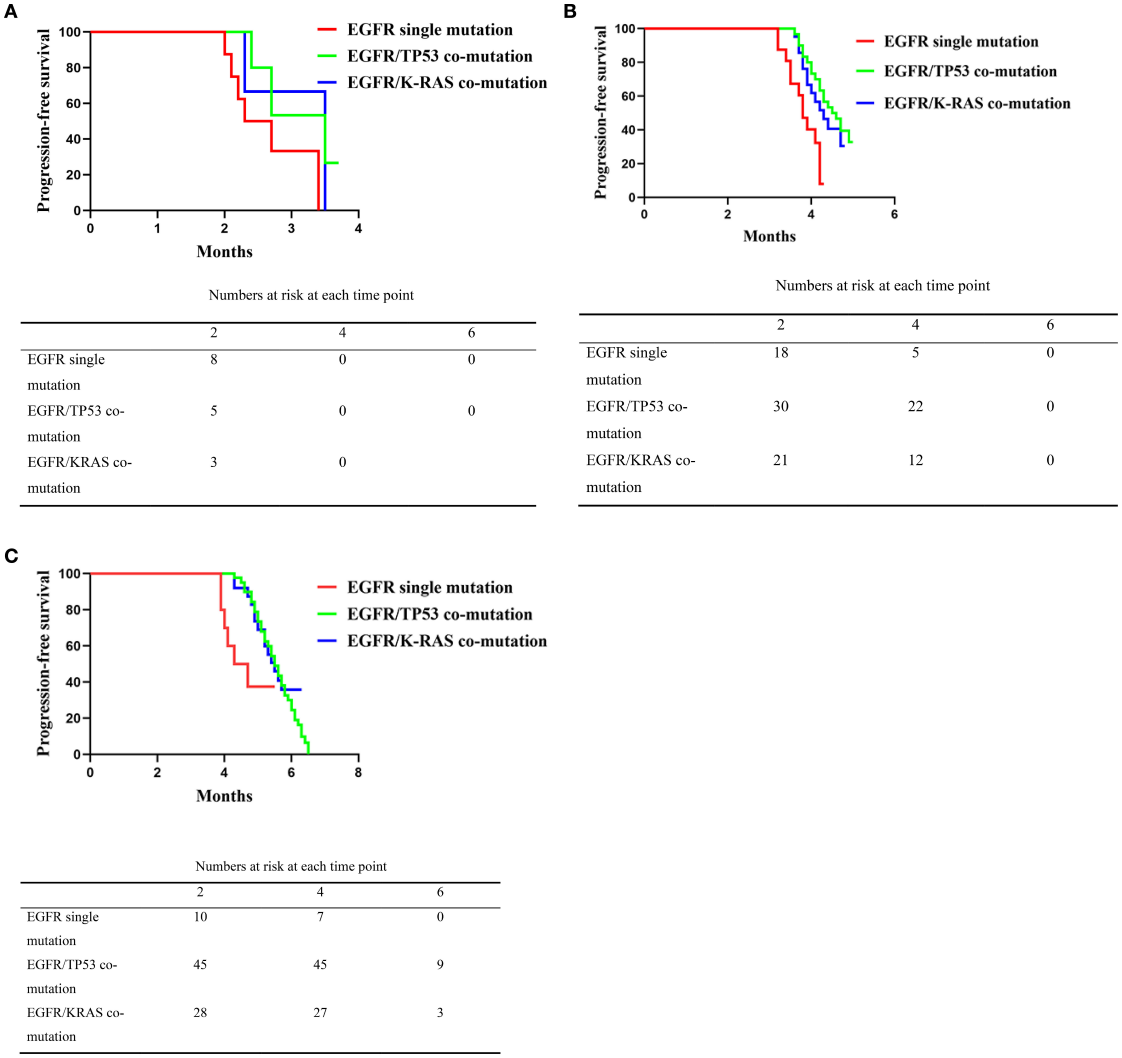
**(A)** Comparison of progression-free survival (PFS) among patients with EGFR single mutation, EGFR/TP53 co-mutation, and EGFR/K-RAS co-mutation in the PD-L1 negative subgroup following second-line platinum-based doublet chemotherapy combined with immunotherapy. The Kaplan–Meier curves show that the EGFR single mutation group had significantly shorter PFS than the co-mutation groups (*P* < 0.0001), while no significant difference was observed between the EGFR/TP53 and EGFR/K-RAS co-mutation groups (*P* = 0.189). **(B)** Comparison of progression-free survival (PFS) among patients with EGFR single mutation, EGFR/TP53 co-mutation, and EGFR/K-RAS co-mutation in the PD-L1 low expression subgroup following second-line platinum-based doublet chemotherapy combined with immunotherapy. The Kaplan–Meier curves show that the EGFR single mutation group had significantly shorter PFS than the co-mutation groups (*P* < 0.0001), with no significant difference between the EGFR/TP53 and EGFR/K-RAS co-mutation groups (*P* = 0.241). **(C)** Comparison of progression-free survival (PFS) among patients with EGFR single mutation, EGFR/TP53 co-mutation, and EGFR/K-RAS co-mutation in the PD-L1 high expression subgroup following second-line platinum-based doublet chemotherapy combined with immunotherapy. The Kaplan–Meier curves indicate that the EGFR single mutation group had significantly shorter PFS than the co-mutation groups (*P* < 0.0001), with no significant difference between the EGFR/TP53 and EGFR/K-RAS co-mutation groups (*P* = 0.312).

Patients were stratified by PD-L1 tumor proportion score (TPS) into negative (<1%), low (1–49%), and high (≥50%) categories. In the PD-L1-negative subgroup, unadjusted Kaplan–Meier analyses showed median PFS of 2.5 months in the EGFR single-mutation group (range, 2.5–3.4), 3.5 months in the EGFR/TP53 group (range, 2.4–3.7), and 3.5 months in the EGFR/KRAS group (range, 2.3–3.8). Pairwise comparisons did not reach statistical significance (*P* = 0.146,95%CI:0.1086-1.503, *P* = 0.246,95%CI:0.1107-1.826, and *P* = 0.646,95%CI:0.2194-9.539) (**Figure 3A**).

In the PD-L1-low subgroup, median PFS was 3.8 months in the EGFR single-mutation group (range, 3.0–4.3), 4.55 months in the EGFR/TP53 group (range, 3.6–5.0), and 4.3 months in the EGFR/KRAS group (range, 3.6–4.8). The single-mutation group differed from the EGFR/TP53 (*P* = 0.0003,95%CI:0.1279-0.7830) and EGFR/KRAS cohorts (*P* = 0.012,95%CI: 0.1770-0.9601), whereas no difference was observed between the EGFR/TP53 and EGFR/KRAS cohorts (*P* = 0.438,95%CI: 0.3693-1.589) (**Figure 3B**).

In the PD-L1–high subgroup, Kaplan–Meier analysis showed a median PFS of 4.5 months in the EGFR single-mutation group (range, 3.9–5.5), 5.5 months in the EGFR/TP53 group (range, 4.1–6.5), and 5.5 months in the EGFR/KRAS group (range, 4.0–6.3). The single-mutation group differed from the EGFR/TP53 (*P* = 0.010,95%CI: 0.3441-1.945) and EGFR/KRAS cohorts (*P* = 0.044,95%CI: 0.3144-2.129), whereas no difference was observed between the EGFR/TP53 and EGFR/KRAS cohorts (*P* = 0.531,95%CI: 0.5380-1.859) (**Figure 3C**).

### 3.5 Multivariate analysis

To assess independent associations with progression-free survival (PFS), we fit multivariable Cox proportional hazards models including prespecified covariates (age, sex, smoking history, histology, clinical stage, ECOG performance status) and mutation status. EGFR/TP53 (HR 1.85, 95% CI 1.22–2.80; *P* = 0.004) and EGFR/KRAS (HR 2.10, 95% CI 1.32–3.33; *P* = 0.002) co-mutations were independently associated with a higher hazard of progression. ECOG PS ≥2 was associated with a numerically higher hazard that did not reach statistical significance (HR 1.45, 95% CI 0.94–2.24; *P* = 0.085). No significant associations were observed for other covariates (all *P*>0.05) (**Table 3**).5.

**Table 3:** Multivariate Cox proportional hazards regression analysis of factors associated with progression-free survival (PFS).

Variable	HR (95% CI)	*P* value
Gene status		
EGFR single mutation	1.00 (reference)	–
EGFR+ TP53 co-mutation	1.85 (1.22–2.80)	0.004
EGFR+K-RAco-mutation	2.10 (1.32–3.33)	0.002
Age (≥65 vs. <65)	1.15 (0.75–1.76)	0.51
Gender (Male vs. Female)	1.09 (0.72–1.65)	0.68
Smoking history	1.23 (0.81–1.87)	0.34
Stage (IV vs. III)	1.37 (0.89–2.13)	0.15
ECOG (≥2 vs. 0–1)	1.45 (0.94–2.24)	0.085
Pathology type (Non-adenocarcinoma vs. Adenocarcinoma)	1.22 (0.82–1.82)	0.31

## 4 Discussion

In EGFR-mutant NSCLC, although EGFR-TKIs generally improve clinical outcomes, there are statistically significant inter-individual differences in progression-free survival (PFS). Consistent with prior literature, our findings indicate that co-mutation status is associated with therapeutic efficacy and immune-related biomarkers, with implications for clinical risk stratification and treatment decision-making (10, 12, 17–23).

### 4.1 Clinical and translational implications of EGFR co-mutations

Prior studies and our data indicate that patients with EGFR/TP53 co-mutations derive limited benefit from EGFR-TKIs and have poorer prognoses (24–26); EGFR/KRAS co-mutations are likewise associated with shorter PFS and an increased risk of resistance (27–29). Taken together, these findings support incorporating co-mutation status into baseline risk assessment at treatment initiation and pre-planning alternative or combination strategies along the therapeutic pathway (10, 12, 28).

### 4.2 Therapeutic implications after resistance to EGFR-TKIs

In the overall EGFR-mutant population, the survival benefit of immunotherapy or platinum-based chemo-immunotherapy appears limited, and pivotal randomized trials (e.g., CheckMate 722 and KEYNOTE-789) did not meet their prespecified primary endpoints (30), underscoring the need for precise patient stratification. In line with our analysis:

- Among patients with EGFR/TP53 co-mutations, second-line chemo-immunotherapy achieved a median PFS of 5.2 months, compared with 3.9 months in those with EGFR mutation alone (P<0.0001), suggesting that TP53 co-mutation may have predictive biomarker potential (31, 32).
- Patients with EGFR/KRAS co-mutations likewise demonstrated a more favorable survival trend on second-line chemo-immunotherapy and frequently exhibited higher PD-L1 levels, consistent with prior reports (33–35).

Taken together, these observations support prioritizing chemo-immunotherapy after EGFR-TKI resistance for patients harboring TP53 or KRAS co-mutations, whereas in patients with isolated EGFR mutations who lack features of immunotherapy sensitivity, immunotherapy-based combinations should be selected with caution (30, 36, 37).

### 4.3 Exploration of anti-angiogenic combinations

Comparing IMpower150 and ORIENT-31 with CheckMate 722 and KEYNOTE-789, a key distinction is that the former two incorporated VEGF inhibitors (30). Given the cross-talk among VEGF signaling, EGFR pathways, and hypoxia responses (38–42), adding anti-angiogenic agents in selected, stratified populations may remodel the tumor microenvironment (TME) and potentiate both immunotherapy and chemotherapy. Accordingly, chemo-immunotherapy with or without anti-angiogenic therapy warrants prospective validation in co-mutated subgroups.

### 4.4 Summary

In the second-line setting after resistance to EGFR-TKIs, we observed a modest prolongation of PFS (approximately 1 month) with platinum-based chemo-immunotherapy in the TP53- or KRAS-co-mutated subgroup. Existing evidence indicates that TP53 mutation is associated with upregulation of immune checkpoints, activation of effector T cells, and increased tumor mutational burden (TMB), whereas KRAS mutation is linked to MAPK-mediated PD-L1 upregulation and neoantigen formation (31–35). Based on these observations, we advance a testable working hypothesis: in EGFR-mutant NSCLC, TP53 or KRAS co-mutation may partially mitigate the “immune-cold” phenotype, thereby enhancing sensitivity to immunotherapy; however, given that pivotal trials such as CheckMate 722 and KEYNOTE-789 did not meet their prespecified primary endpoints (30), this should be regarded as hypothesis-generating rather than confirmatory and requires validation in functional studies and prospective trials.

Differences between our findings and randomized data may reflect heterogeneity in study populations and treatment regimens—including prior TKI generation and exposure, crossover, eligibility criteria, and biomarker distributions. Accordingly, prospective multi-center studies with pre-specified stratification and interaction analyses by co-mutation profile and immune-related biomarkers (e.g., PD-L1, TMB, and TME features), alongside head-to-head comparisons with contemporary standards, are warranted to determine whether co-mutated subgroups derive reproducible and durable benefit from second-line chemo-immunotherapy.

## Data Availability

The raw data supporting the conclusions of this article will be made available by the authors, without undue reservation.
